# Alpinetin Alleviates Cardiac Inflammation and Remodeling via TLR4/MyD88/NF-κB Signaling Pathway in Rats with Acute Myocardial Infarction

**DOI:** 10.3390/ijms262010073

**Published:** 2025-10-16

**Authors:** Mei Feng, Xinxiang Chen, Fan Huang, Lin Chen, Can Liu, Wei Li, Yinyan Li, Shaobin Chen, Zhen Deng, Zhengyi Wei, Yuan Luo, Xiyong Yu, Aiping Qin

**Affiliations:** 1 Guangzhou Municipal and Guangdong Provincial Key Laboratory of Molecular Target & Clinical Pharmacology, The NMPA and State Key Laboratory of Respiratory Disease, School of Pharmaceutical Sciences, Guangzhou Medical University, Guangzhou 511436, China2023211626@stu.gzhmu.edu.cn (X.C.); 13715607546@163.com (W.L.);; 2 School of Basic Medical Sciences, Guangzhou Medical University, Guangzhou 511436, China

**Keywords:** alpinetin, acute myocardial infarction, inflammation, remodeling, NF-κB signaling pathway

## Abstract

Alpinetin, a distinctive plant-derived dihydroflavonoid from cardamom seeds, represents an under-explored chemical scaffold compared to common flavonoids like quercetin or kaempferol. While many flavonoids have shown general cardioprotective potential, the structural specificity of alpinetin may confer unique pharmacological advantages. Inspired by its historical use in traditional Chinese medicine for cardiac discomfort, this study systematically investigated its efficacy against acute myocardial infarction (AMI). In a rat AMI model, alpinetin demonstrated superior infarct size reduction and functional recovery relative to other tested flavonoids. It significantly attenuated key AMI pathologies—including inflammatory infiltration, CD68+ macrophage activation, IL-6/TNF-α release, collagen deposition, and cardiomyocyte apoptosis—more effectively than common flavonoid benchmarks. Mechanistically, alpinetin selectively targeted the TLR4/MyD88/NF-κB signaling axis with notable potency, a pathway not robustly modulated by other flavonoids in the screening. These findings not only validate the traditional wisdom of cardamom but also establish alpinetin as a structurally and mechanistically distinct flavonoid with high translational promise, offering a new candidate for the targeted treatment of ischemic heart disease.

## 1. Introduction

Acute myocardial infarction (AMI) is a prevalent cardiovascular disease that has attracted considerable attention. Inflammation, necrosis, and cardiac insufficiency, resulting from myocardial infarction, eventually lead to congestive heart failure [1]. Studies have shown that treatment for AMI focuses on controlling infarct size, regulating myocardial remodeling, and preventing heart failure. In particular, the inflammatory response plays a crucial role in myocardial injury, repair, and remodeling [2]. Therefore, regulating myocardial inflammation has become an important target in the treatment of AMI.

In recent years, plant-derived flavonoids have attracted significant research interest owing to their natural origins, potent pharmacological activities, and favorable safety profiles compared to synthetic drugs [3,4,5]. Flavonoids encompass a wide range of structurally diverse compounds, which are classified into several subgroups—including flavones, flavonols, dihydroflavones, dihydroflavonols, isoflavones, chalcones, anthocyanins, and flavanols—based on their chemical skeletons. Importantly, structural differences strongly influence their biological activities, leading to distinct functional properties among various subclasses [6,7,8].

Alpinetin (ALP), a distinctive dihydroflavone derived from cardamom seeds, represents a structurally unique flavonoid with promising pharmacological potential. Like other well-studied flavonoids, alpinetin has demonstrated broad anti-inflammatory effects in vitro, in vivo, and in clinical settings [9,10,11]. Its relevance to cardiac protection is suggested by the traditional Chinese medicine classic Ben Cao Feng Yuan, which notes that cardamom—the natural source of alpinetin—is “warm in nature and appropriate for those suffering from pain in the cardiac region.” While several flavonoids such as quercetin [12], luteolin [13], and baicalin [14] have shown cardioprotective properties, these compounds belong to the flavonol or flavone subclasses, whereas alpinetin is a dihydroflavone. This structural distinction implies potential differences in mechanism and efficacy, underscoring the need to explore the structure–activity relationship of alpinetin in the context of acute myocardial infarction (AMI). Therefore, this study aimed to systematically evaluate the therapeutic effects and underlying mechanisms of alpinetin in an AMI rat model.

## 2. Results

### 2.1. ALP Reduced Myocardial Injury in AMI Rats

**Figure 1:**
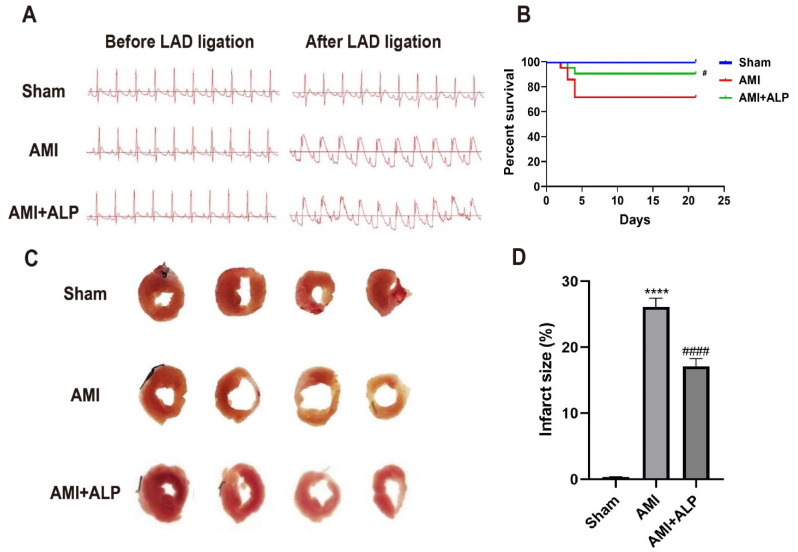
ECG and cross-sections of the hearts in three groups. (**A**) ECG changes before and after LAD ligation. (**B**) Survival analysis in the 21 days following AMI. *n* = 16. (**C**) Morphological changes in the cross-sections of rat hearts 21 days after AMI. (**D**) Statistical results of the percentage in left ventricular infarction. *n* = 6. (**** *p* < 0.0001 vs. Sham; ^#^ *p* < 0.05, ^####^ *p* < 0.0001 vs. AMI).

To confirm the successful establishment of the AMI model, ECG changes were assessed before and after LAD ligation. As shown in Figure 1A, myocardial ischemia was confirmed by regional cyanosis and ST-segment elevation in the AMI and AMI+ALP groups, in comparison to the Sham group. At 21 days after AMI, the rats’ hearts were isolated, revealing an obvious white infarcted area with thin ventricular walls and a clear boundary in the cardiac cross-sections induced by AMI. In contrast, ALP treatment significantly ameliorated these morphological changes (Figure 1C,D). In addition, ALP treatment considerably improved the survival period of rats at 21 days after AMI (Figure 1B). These results demonstrated that ALP effectively reduced AMI-induced myocardial injury and improved the survival rate.

### 2.2. ALP Reduced Myocardial Remodeling in AMI Rats

**Figure 2:**
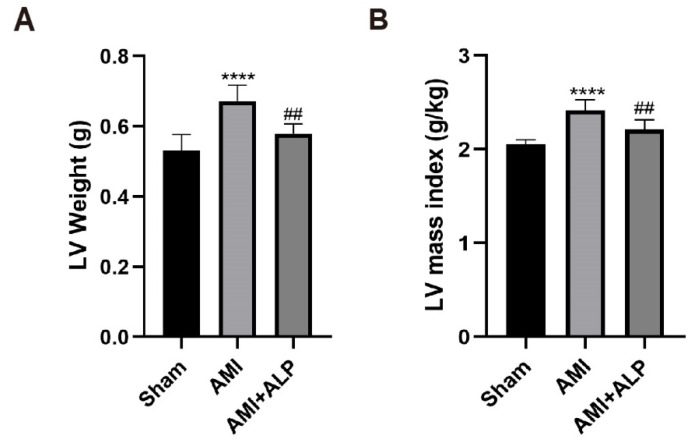
Cardiac indices of rats in the three groups 21 days after AMI. (**A**) LV weight. (**B**) LV mass index. Data are expressed as the means ± SD, *n* = 6. (**** *p* < 0.0001 vs. Sham; ^##^ *p* < 0.01 vs. AMI).

To investigate the effect of ALP on myocardial remodeling, the cardiac indexes 21 days after AMI were evaluated. As shown in Table 1 and Figure 2, body weights and heart weights have no significant difference among the three groups of rats. However, the left ventricular (LV) weight and LV mass index were significantly higher than AMI rats 21 days after AMI, compared to the Sham group. After ALP treatment, LV weight and LV mass index caused by AMI significantly decreased. The results indicated that ALP attenuated myocardial remodeling induced by AMI.

### 2.3. ALP Ameliorated Cardiac Dysfunction in AMI Rats

**Figure 3:**
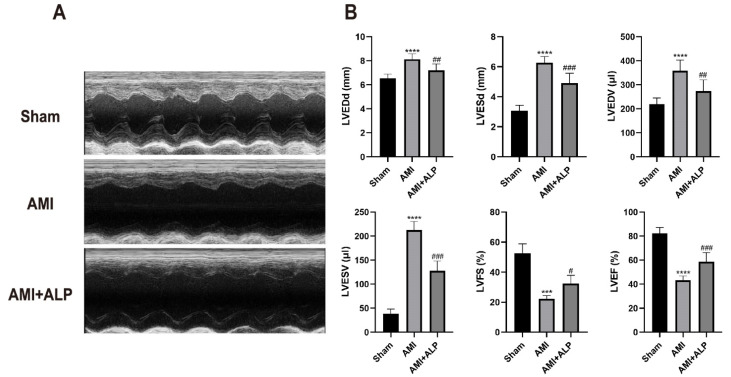
Echocardiography (UCG) (ultrasonic cardiogram) changes in the three groups 21 days after AMI. (**A**) Representative UCG images. (**B**) Analysis of UCG results. LVEDd, left ventricular end-diastolic dimension; LVESd, left ventricular end-systolic dimension; LVEDV, left ventricular end-diastolic volume; LVESV, left ventricular end-systolic volume; LVFS, left ventricular fractional shortening; LVEF, left ventricular ejection fraction. Data were analyzed using one-way ANOVA. Data are expressed as the means ± SD, *n* = 6. (*** *p* < 0.001, **** *p* < 0.0001 vs. Sham; ^#^ *p* < 0.05, ^##^ *p* < 0.01, ^###^ *p* < 0.001 vs. AMI).

Echocardiography was employed to assess cardiac function 21 days post-AMI. Rats in the AMI group exhibited significant left ventricular dysfunction, characterized by a marked increase in left ventricular end-diastolic and end-systolic dimensions (LVEDd, LVESd) and volumes (LVEDV, LVESV), along with a pronounced reduction in left ventricular fractional shortening (LVFS) and ejection fraction (LVEF). Systolic function was evaluated using LVEF, LVFS, LVESd, and LVESV, while diastolic function was assessed via LVEDd and LVEDV. Treatment with ALP significantly ameliorated these AMI-induced functional impairments. Specifically, ALP administration led to a notable improvement in systolic parameters, as reflected by the recovery of LVEF and LVFS, together with a reduction in LVESd and LVESV. These findings indicate that ALP treatment effectively attenuates AMI-induced myocardial dysfunction, with a particularly beneficial impact on systolic performance (Figure 3).

### 2.4. ALP Suppressed the Inflammatory Response in the Hearts of AMI Rats

**Figure 4:**
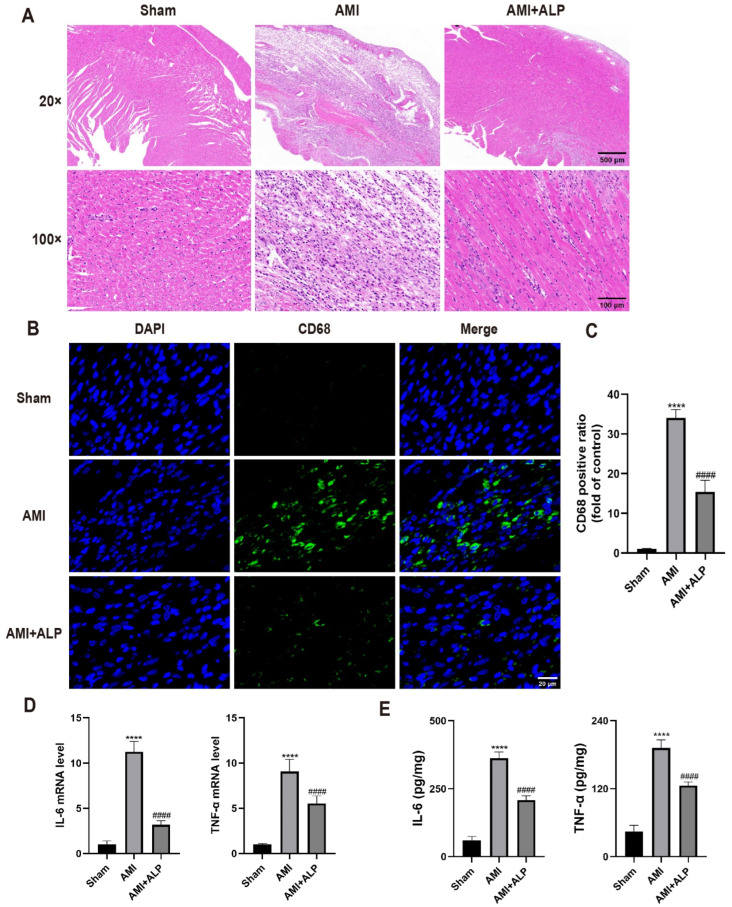
Anti-inflammatory effects of Alpinetin on AMI rats. (**A**) Pathological changes in rat hearts by H&E staining in three groups 3 days after AMI (20× magnification, scale bar = 500 μm, 100× magnification, scale bar = 100 μm). (**B**,**C**) CD68+ cells were detected by immunofluorescence staining 3 days after AMI (**B**) (400× magnification, scale bar = 20 μm). Quantitative analysis of CD68 cells (**C**). (**D**,**E**) The expression of pro-inflammatory cytokines IL-6 and TNF-α in the rat myocardial tissue was analyzed 3 days after AMI by qRT-PCR (**D**) and ELISA (**E**). Data are expressed as the means ± SD, *n* = 6. (**** *p* < 0.0001 vs. Sham; ^####^ *p* < 0.0001 vs. AMI).

To assess the effects of ALP on AMI-induced myocardial inflammation, the histopathological changes in the rat hearts by H&E staining were examined. Compared to the Sham group, the rats in the AMI group exhibited disrupted myocardial architecture, loss of cardiomyocyte, extensive inflammatory infiltration, interstitial hemorrhage, cell light staining, and perinuclear vacuole (Figure 4A). In addition, H&E staining revealed granular degeneration of myocardial fibers and fiber fractures in AMI rats. Conversely, ALP treatment significantly reduced the infiltration of inflammatory cells in the infarct area, and the arrangement of cardiomyocytes was more orderly than that in AMI rats. The results were further confirmed by quantifying the number of myocardial CD68 cells using immunofluorescence assay. A significant reduction in CD68 expression was detected after ALP treatment, compared to the AMI group (Figure 4B,C).

As inflammatory cytokines play an important role in the pathogenesis of cardiovascular diseases [15], the levels of inflammatory cytokines in rats’ myocardial tissue at three days after AMI by ELISA were detected. The results showed that ALP treatment significantly decreased the expression of pro-inflammatory cytokine IL-6 and TNF-α at the mRNA and protein levels compared to AMI rats (Figure 4D,E). The above results demonstrated that ALP treatment significantly reduced myocardial inflammatory of AMI rats.

### 2.5. ALP Alleviated the Collagen Deposition in AMI Rats

**Figure 5:**
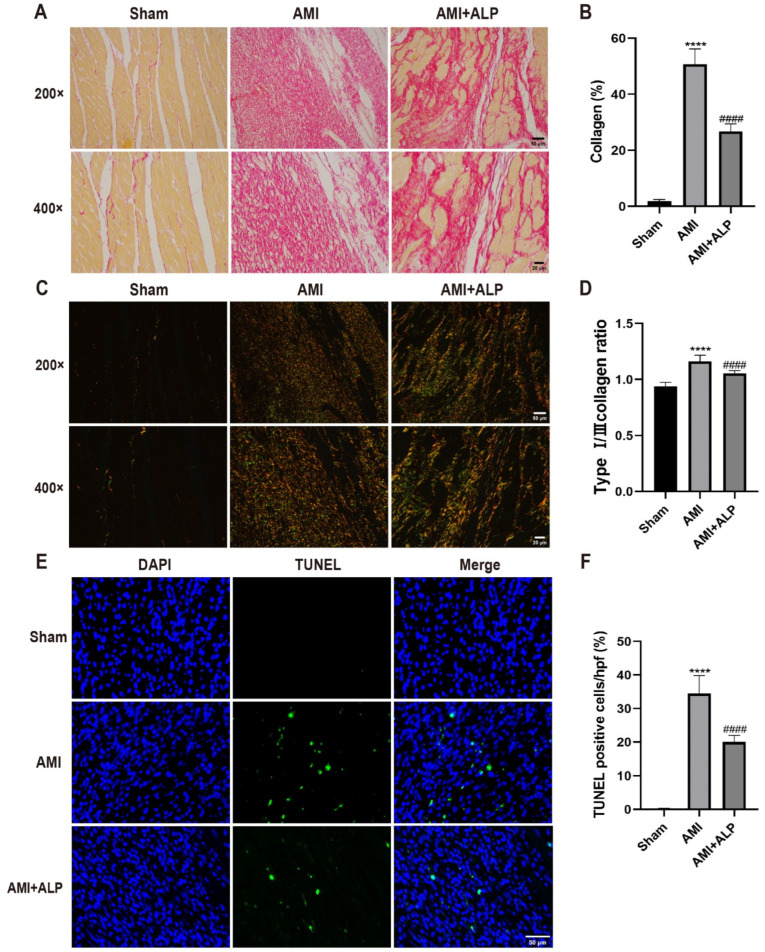
Pathological changes in the rat hearts after AMI. (**A**) Images under white light. (**B**) Collagen areas of the hearts. (**C**) Images under polarized light. (Orange yellow: type I collagen, Green: type III collagen fiber). (**D**) Proportion of type I/III collagen of the hearts. (200× magnification, scale bar = 50 μm, 400× magnification, scale bar = 20 μm). (**E**) TUNEL assay in border region of hearts 3 days after AMI, stained for Tunel (green). Nuclei were stained with DAPI (blue) (200× magnification, Scale bar = 50 μm). (**F**) Quantitative analysis of apoptotic cells. Data are expressed as the means ± SD, *n* = 6. (**** *p* < 0.0001 vs. Sham; ^####^ *p* < 0.0001 vs. AMI).

Persistent myocardial inflammation could result in collagen deposition and an imbalance in the ratio of type I/III collagen, particularly an increase in type I/III collagen, affecting cardiac systolic and diastolic functions [16]. Quantitative analysis of Sirius Red staining revealed myocardial collagen deposition areas of 4.77 ± 1.06%, 49.06 ± 3.32%, and 30.94 ± 4.20% in the Sham group, the AMI group, and the AMI+ALP group, respectively. ALP treatment markedly decreased myocardial collagen deposition at 21 days, compared to the AMI rats (Figure 5A,B). A significant decrease in the ratio of type I/III collagen within the infarct zone was also observed following ALP treatment (Figure 5C,D). Additionally, the effect of ALP on cardiomyocyte apoptosis at three days after AMI by using TUNEL staining was evaluated. The results showed that TUNEL-positive cells significantly decreased compared with the AMI rats (Figure 5E,F). These data illustrated that ALP alleviated myocardial fibrosis and cardiomyocyte apoptosis induced by AMI.

### 2.6. ALP Downregulated the Expressions of TLR4 and MyD88 in AMI Rats

**Figure 6:**
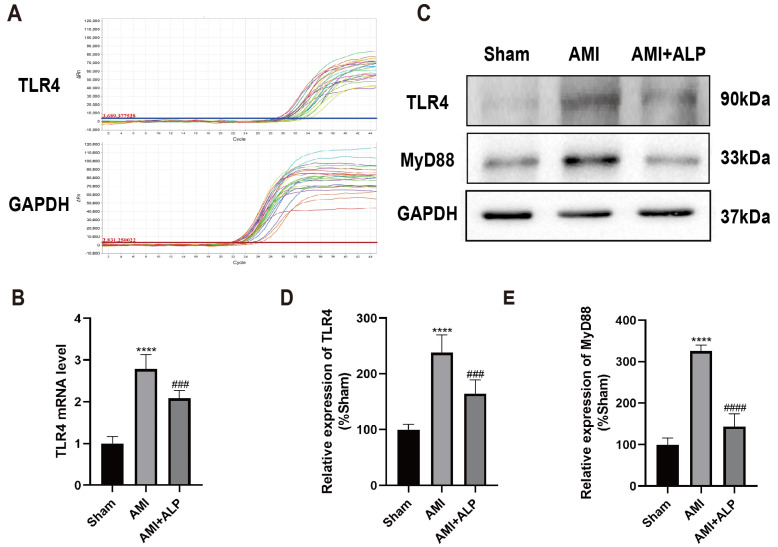
Expressions of TLR4 and MyD88 in rat hearts. (**A**) TLR4 mRNA amplification curve by qRT-PCR 21 days after AMI. (**B**) TLR4 mRNA levels. (**C**) Representative Western blotting images of TLR4 and MyD88 protein. (**D**) Quantitative analysis of TLR4 protein. (**E**) Quantitative analysis of MyD88 protein 21 days after AMI. Data are expressed as the means ± SD, *n* = 6. (**** *p* < 0.0001 vs. Sham; ^###^ *p* < 0.001, ^####^ *p* < 0.0001 vs. AMI).

To further investigate the mechanisms of ALP’s protection against AMI, the possible signal pathways involved were explored. As primary receptors of innate immunity, toll-like receptors (TLRs) could initiate innate immune defense by interacting with pro-inflammatory pathways, leading to the development and deterioration of inflammatory diseases. Previous studies have found that TLR4 plays a crucial role in mediating acute myocardial dysfunction caused by myocardial ischemia [17]. So, the level of TLR4 from cardiac LV 21 days after AMI using qRT-PCR was first detected. As shown in Figure 6A,B, a significant increase in TLR4 expression in the myocardium of AMI rats could be observed, whereas ALP treatment significantly diminished the expression of TLR4. These results were further confirmed by Western blotting analysis (Figure 6C,D). Meanwhile, after ALP treatment, a significant decrease in the expression of MyD88, an important adaptor molecule of TLR4, could also be found (Figure 6C,E). These results suggested that ALP treatment could effectively alleviate myocardial inflammation caused by AMI via TLR4/MyD88 signaling pathways.

### 2.7. ALP Inhibited the Phosphorylation of IκBα and NF-κB p65 Protein in Myocardium of AMI Rats

**Figure 7:**
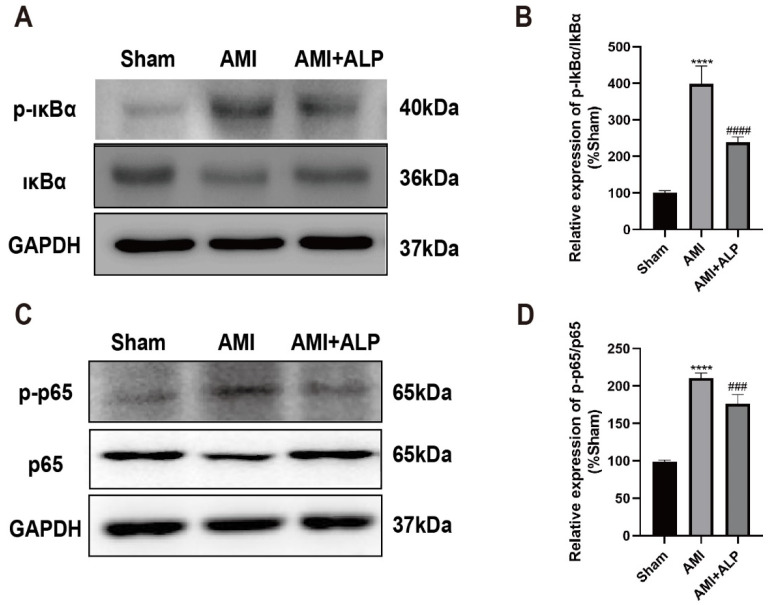
Expressions of p-IκBα, IκBα, NF-κB p-p65, and p65 proteins in rat hearts by Western blotting 21 days after AMI. (**A**) Representative images of p-IκBα and IκBα proteins. (**B**) Quantitative analysis of IκBα and p-IκBα protein. (**C**) Representative images of p-p65 and p65 proteins. (**D**) Quantitative analysis of p65 and p-p65 protein. Data are expressed as the means ± SD, *n* = 6. (**** *p* < 0.0001 vs. Sham; ^###^ *p* < 0.001, ^####^ *p* < 0.0001 vs. AMI).

**Figure 8:**
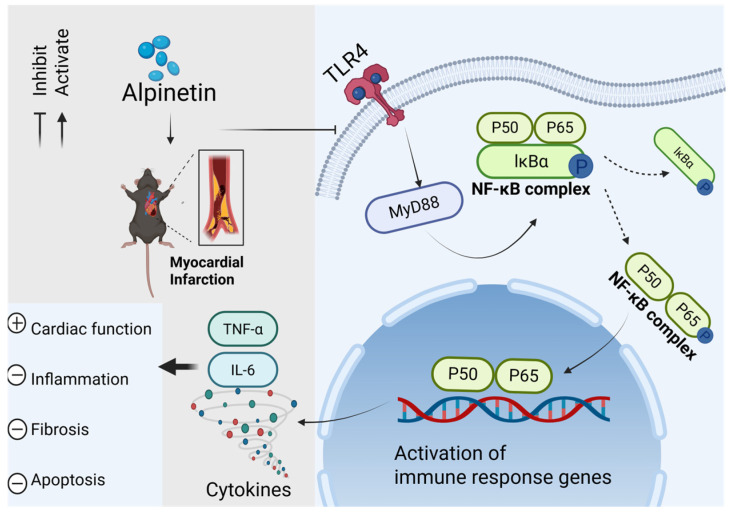
Proposed working model for the cardioprotective effects of alpinetin in AMI. Alpinetin (ALP) protected rats from AMI injury by reducing inflammation of infarction sites and myocardial fibrosis via inhibiting the TLR4/Myd88/NF-κB pathway. Cell death caused by AMI in the infarct region recruits and activates immune cells, which mount an inflammatory response in the early phase. TLR4 is one of the most highly expressed in the context of myocardial injury, and its activation recruits MyD88 adaptor proteins with complementary binding interfaces. This combination promotes cytoplasmic ubiquitination and degradation of the NF-κB inhibitor IκBα, leading to phosphorylation of the NF-κB subunit p65/p50 isodimer, its nuclear translocation, and the transcription of various inflammatory genes, such as TNF-α and IL-6. ALP treatment restricts this process, such as reduced cardiac repair, scar formation, cardiac fibrosis, and apoptosis. This working model summarizes the proposed mechanism by which alpinetin attenuates AMI-induced injury. Solid arrows represent pathways or components supported by direct experimental evidence from this study. Dashed arrows indicate postulated connections based on the established literature and observed phenotypic outcomes.

Given that MyD88 can activate NF-κB, a key transcription factor involved in inflammation, the phosphorylation levels of IκBα and NF-κB p65 in the myocardium of AMI rats were assessed using Western blotting [18,19]. As shown in Figure 7A–D, ALP treatment significantly reversed the phosphorylation of IκBα and NF-κB p65 induced by AMI 21 days after AMI, compared to the AMI rats. These results revealed that the protective effects of ALP against AMI involve the inhibition of the TLR4/MyD88/NF-κB signaling pathway (Figure 8).

## 3. Discussion

This study provides the first systematic evaluation of ALP, a unique dihydroflavone from Alpinia katsumadai Hayata, in AMI. While flavonoids are known for their anti-inflammatory properties [20], and ALP itself has demonstrated efficacy in the respiratory, digestive, reproductive, and motor systems [9,10,11,21,22,23,24], its role in cardiovascular disease remained unclear. Our findings establish that ALP confers comprehensive cardioprotection by improving cardiac function, attenuating inflammation, and suppressing fibrosis post-AMI, mechanistically linked to modulation of the TLR4/MyD88/NF-κB pathway.

ALP treatment prompted substantial functional recovery by day 21 post-AMI. This was evidenced through reduced infarct size (Figure 1), decreased left ventricular weight and mass index (Figure 2), and improved echocardiographic parameters. Specifically, ALP enhanced LVEF and LVFS, while reducing left ventricular end-systolic and end-diastolic dimensions and volumes (LVESd, LVEDd, LVESV, LVEDV) (Figure 3). These results indicate ALP mitigates acute injury and ameliorates adverse ventricular remodeling.

Excessive inflammation is a key driver of myocardial injury after AMI [25,26]. ALP treatment significantly attenuated inflammatory cell infiltration, particularly CD68+ macrophages, and suppressed myocardial levels of IL-6 and TNF-α (Figure 4). This anti-inflammatory effect aligns with clinical strategies to curb early excessive inflammation, as supported by trials like CANTOS and COLCOT [27,28], though the timing of the intervention is critical given the dual role of inflammation in injury and repair [29].

Mechanistically, ALP downregulated TLR4 and MyD88 expression, inhibited IκBα and NF-κB phosphorylation, and consequently reduced pro-inflammatory gene expression (Figure 6 and Figure 7). TLR4/MyD88 activation promotes IκBα degradation, enabling NF-κB nuclear translocation and transcription of inflammatory genes [30,31,32,33,34]. Elevated cardiac TLR expression is linked to pro-inflammatory responses and contractile dysfunction [35,36], while sustained TNF-α and IL-6 elevation aggravates remodeling and fibrosis [37,38]. Thus, ALP ameliorates cardiac inflammation primarily by inhibiting the TLR4/MyD88/NF-κB axis [39,40].

Chronic inflammation promotes fibroblast activation and fibrosis [41,42]. ALP significantly reduced collagen deposition and, crucially, lowered the pathological ratio of type I to type III collagen. Type I collagen confers stiffness to fibrotic scars, whereas type III collagen contributes to myocardial elasticity [43]. Excessive type I collagen deposition increases myocardial stiffness and dysfunction [44,45,46]. ALP’s favorable modulation of collagen composition likely underlies improved myocardial compliance. This effect may stem from the suppression of the inflammatory TLR4/NF-κB pathway, a key upstream stimulator of fibrotic processes, and potentially through inhibition of the pro-fibrotic TGF-β1/Smad pathway, as reported in other models [47].

The superiorities of this research are threefold. First, it focuses on ALP, a structurally distinct dihydroflavonoid previously overlooked in cardiovascular research, unlike common flavones or flavonols. Second, it provides a comprehensive assessment from whole-organ function to molecular mechanism. Third, it identifies a specific and potent inhibitory effect on the TLR4/MyD88/NF-κB pathway, a mechanism not robustly reported for other flavonoids, highlighting ALP’s unique profile.

A key limitation is the unresolved cellular specificity of ALP’s action. Future studies should employ cell-type-specific models to identify primary targets (e.g., macrophages, cardiomyocytes, fibroblasts). Additionally, while anti-inflammatory effects are central, other pathways may contribute, such as ALP’s reported vasodilatory effect [48,49], which could reduce cardiac afterload and synergize with direct myocardial effects. Monitoring systemic hemodynamics and employing techniques like electrophoretic mobility shift assays for NF-κB DNA-binding activity will further delineate ALP’s mechanisms.

In summary, these findings establish ALP as a structurally and mechanistically distinct flavonoid with high translational promise. By demonstrating its efficacy in ameliorating dysfunction, inflammation, and fibrosis post-AMI via targeted inhibition of the TLR4/MyD88/NF-κB pathway, this study validates its traditional use and offers a compelling candidate for targeted treatment of ischemic heart disease.

## 4. Materials and Methods

### 4.1. Reagents

ALP was purchased from Aladdin (Shanghai, China). CMC-Na (Sigma, St. Louis, MO, USA), Anti-TLR4 (19811-1), anti-IκBα (10268-1), anti-p65 (10745-1), and anti-GAPDH (60004-1) were from Proteintech (Chicago, IL, USA). Anti-Phospho-IκBα (ab133462), anti-Phospho-p65 (ab16502), and rabbit anti-MyD88 (ab219413) were obtained from Abcam Biotech (Cambridge, UK). BCA Protein Assay Kit and Horseradish-peroxidase labeled goat anti-rabbit IgG were purchased from Beyotime Biotechnology (Shanghai, China). Trizol was from Invitrogen (Carlsbad, CA, USA). RevertAid™ First Strand cDNA Synthesis Kit and AceQ^TM^qPCR SYBR^®^ Green Master Mix were obtained from Vazyme (Nanjing, China). Rat TNF-α and IL-6 ELISA Kit were purchased from NeoBioscience Technology Co., Ltd. (Shenzhen, China).

### 4.2. Alpinetin Preparation

Alpinetin (purity > 98%, Cat. No.: A357136, Aladdin) was dissolved daily in a vehicle consisting of 10% dimethyl sulfoxide (DMSO) and 0.5% carboxymethyl cellulose sodium (CMC-Na) in 0.9% saline. The AMI group received an equal volume of the vehicle alone (10% DMSO + 0.5% CMC-Na) to control for potential effects of the solvent.

### 4.3. Animals and Experimental Protocols

All animal experiments were conducted in compliance with the ARRIVE guidelines and approved by the institutional Animal Care and Use Committee of Guangzhou Medical University (Approval No.: GY2021-057, GY2024-714). The exact number of animals (biological replicates, n) used for each experiment is explicitly stated in the respective figure legends and the Section 4 as follows: survival study: *n* = 16 per group; TTC staining, Echocardiography, H&E staining, Immunofluorescence, qRT-PCR, ELISA, Sirius Red staining, and TUNEL assay, *n* = 6 per group.

Adult male Sprague-Dawley rats (10 weeks old, weighing approximately 230–270 g) were sourced from the Medical Experimental Animal Center of Guangdong Province and acclimatized for one week in advance in the SPF animal facility. All rats were placed in a room maintained at a temperature of 25 ± 2 °C and a humidity of 55 ± 5% with free access to standard food and water for 12 h [50,51].

AMI was induced as previously described [52]. In brief, the rats were anesthetized with 1% pentobarbital sodium (30 mg/kg, ip) and mechanically ventilated using an animal respirator. Myocardial ischemia was induced by completely ligating the left anterior descending (LAD) coronary artery. Briefly, after anesthesia and ventilation, a left thoracotomy was performed at the 4th intercostal space. The LAD was ligated 2 mm distal to the left atrial appendage using a 6-0 Prolene suture, ensuring the full-thickness ligation of the vessel. Successful occlusion was confirmed intraoperatively by the immediate blanching of the anterior left ventricular wall and ST-segment elevation on ECG. Postoperatively, at 24 h, echocardiography was used to confirm significant left ventricular dysfunction (LVEF < 45% in the AMI group versus > 65% in the Sham group). Rats not meeting this functional criterion (exclusion rate ~10%) were excluded from the study. Rats that survived 24 h post-thoracotomy were then divided into three groups as follows: (1) Sham group (*n* = 22): animals underwent the same surgical procedure but without LAD ligation; (2) AMI group (*n* = 22): rats received LAD ligation and the same volume of saline alone; (3) AMI + ALP group (*n* = 22). Rats were administered ALP (20 mg/kg/d, intragastrically) starting 24 h after AMI induction and continuing for 21 days.

### 4.4. Echocardiogram (UCG)

Echocardiography was performed on day 21 post-AMI under anesthesia induced with 2% isoflurane at a flow rate of 2.5 mL/min. Cardiac function was assessed using a Vevo^®^ 2100 Imaging System (VisualSonics Inc., Toronto, ON, Canada) equipped with a 13–24 MHz transducer. All image acquisition and analyses were conducted in a double-blinded manner, where the operator was unaware of group assignments during scanning, and a second blinded investigator analyzed the coded images. The group code was revealed only after the final analysis was complete. Both parasternal long-axis and short-axis views of the left ventricle were obtained. Key parameters of the left ventricular function, including LVEF, LVEDV, and LVESV, were measured over 3 to 5 consecutive cardiac cycles, and the mean value was used as the final result for each animal.

### 4.5. Cardiac Mass Index

Cardiac samples were collected 21 days after AMI. Rats were euthanized via intraperitoneal injection of a lethal of pentobarbital. The cardiac tissue was rinsed with pre-cooled PBS and dried with filter paper, and the left ventricle was then isolated. Body weight (BW), heart weight (HW) and left ventricular weight (LVW) were recorded, and the LVW/BW (for LV mass index) and HW/BW (for Cardiac Mass Index) ratios were calculated to evaluate the degree of cardiac remodeling.

### 4.6. Histopathological Examination

Heart samples were embedded in paraffin, sectioned into 4 μm thick slices, and stained with hematoxylin and eosin (H&E). Myocardial fibrosis was quantified from Sirius Red-stained sections [53]. Tissue sections were imaged using a microscope (Eclipse CI-L, Nikon, Tokyo, Japan). The fibrotic area was calculated as (Area of red staining/Total tissue area) × 100%. The final value for each animal represents the mean percentage from the three sections. All quantifications were performed by an investigator blinded to the group allocation. The collagen area and the ratio of type I/III collagen were quantified using Image-Pro Plus 6.0 (Media Cybemetics, Rockville, MD, USA). For immunofluorescence, tissue sections were labeled with primary antibodies followed by Alexa Flour 488- or Alexa Flour 555-conjugated secondary antibodies (Invitrogen). Antibodies against CD68 (1:50, sc20060) were purchased from Santa Cruz Biotechnology (Dallas, TX, USA). For TUNEL staining, tissue sections were used to detect the death of cardiomyocytes at the border region of the infarcted heart using the TUNEL staining fluorescence detection kit (Beyotime Institute of Biotechnology, Shanghai, China). DAPI (MedChemExpress, Monmouth Junction, NJ, USA) staining was performed to identify the nuclei. TUNEL-positive cells were observed under a fluorescent microscope (Leica, Buffalo Grove, IL, USA) at 200× magnification and analyzed using Zen imaging software (version 3.9) or ImageJ software (version 1.53).

### 4.7. Quantitative Real-Time PCR (qRT-PCR) Detection

Total RNA was isolated from the left ventricular (LV) tissue using the SteadyPure Universal RNA Extraction Kit II (Accurate Biotechnology (HUNAN) Co., Ltd., Changsha, China) according to the manufacturer’s protocol. RNA concentration and purity were determined spectrophotometrically (NanoDrop, Thermo Fisher Scientific, Waltham, MA, USA), with all samples exhibiting an A260/A280 ratio of approximately 1.9. First-strand cDNA was synthesized from total RNA using the HiScript 1st Strand cDNA Synthesis Kit. Quantitative real-time PCR (qRT-PCR) was then performed on an Applied Biosystems ViiA 7^TM^ system using AceQ^TM^qPCR SYBR^®^ Green Master Mix. Each sample was run in technical triplicates. To ensure data reliability, the following validation steps were implemented: (1) primer specificity was confirmed by melt curve analysis, which yielded a single peak for each amplicon, and (2) GAPDH was validated as a stable reference gene, as its expression did not vary significantly across experimental groups. Relative gene expression was calculated using the 2(−ΔΔCt) method. Primer sequences were as follows:

TLR4, F—5′-TCTGCCCTGCCACCATTTACA-3′, R—5′-GTCCCAGCCAGATGCAAGAGA-3′; IL-6, F—5′-GCCCACCAGGAACGAAAGTC-3′, R—5′-TGGCTGGAAGTCTCTTGCGG-3′; TNF-α, F—5′-GATCGGTCCCAACAAGGAGG-3′, R—5′-CTTGGTGGTTTGCTACGACG-3′; GAPDH, F—5′-CGGCAAGTTCAACGGCACAGT-3′, R—5′-CGACATACTCAGCACCAGCATCAC-3′.

### 4.8. Western Blotting Analysis

Total protein was extracted from cardiac LV, and the protein concentration was measured. A total of 40 μg of protein was separated by 10% SDS-Page electrophoresis and transferred to NC membrane. The membranes were incubated in 5% fat-free milk at room temperature for 2 h and then probed with primary antibodies at 4 °C overnight. The primary antibodies TLR4, MyD88, p65, p-p65, IκBα, p-IκBα were diluted to 1:1000, respectively, and GADPH to 1:5000. After washing with 10% TBST for five times, the membranes were incubated with a 1:5000 dilution of goat anti-rabbit at room temperature for 1 h. After 3-4 times of washing in TBST (each time for 10 min), blots were taken to the autoradiography by ECL reagents (BeyoECL Plus P0018S, Beyotime Biotechnology, Shanghai, China), and the signals were detected using an automatic chemiluminescence image analysis system (Tanon 4600, Shanghai, China). Protein levels were normalized to GAPDH. Band intensities for target proteins were quantified using ImageJ and expressed as the ratio of target protein intensity to GAPDH intensity. Data are from *n* = 6 biological replicates per group.

### 4.9. Enzyme-Linked Immunosorbent Assay (ELISA)

After homogenate from cardiac LV were collected, the concentration of IL-6 and TNF-α are detected by ELISA kits (NeoBioscience Technology Co., Ltd., Shenzhen, China) in accordance with the manufacturer’s instructions. The myocardial infarction tissue was weighed and prepared into a 10% homogenate. Cytokine levels in tissue homogenates are expressed as pg/mg. Serum cytokine levels are expressed directly as pg/mL. Data are from *n* = 6 biological replicates per group.

### 4.10. Statistical Analysis

The data were presented as mean ± SD of three independent experiments. Group differences were analyzed using one-way analysis of variance (ANOVA) with Tukey’s post hoc test. *p* < 0.05 was considered statistically significant. ** represents *p* < 0.01, *** represents *p* < 0.001, and **** represents *p* < 0.0001 compared with the Sham group; ^#^ represents *p* < 0.05, ^##^ represents *p* < 0.01, ^###^ represents *p* < 0.001, and ^####^ represents *p* < 0.0001 compared with the AMI group. The sample size for key endpoints was determined based on pilot studies and power analysis to ensure a power of >80% with an alpha of 0.05. The normality of data distribution for all quantitative datasets was verified using the Shapiro–Wilk test before applying parametric statistical tests.

## 5. Conclusions

In conclusion, this study addresses a critical knowledge gap by demonstrating that ALP, a distinctive dihydroflavonoid with a history of traditional use, confers significant cardioprotection against AMI through a mechanism distinct from common flavonoids. This study showed that its potent efficacy is mediated, at least in part, by the selective suppression of the TLR4/MyD88/NF-κB signaling axis, leading to a comprehensive attenuation of inflammation, fibrosis, and apoptosis.

However, the limitations of this current work should be acknowledged. The mechanistic insights are primarily derived from in vivo observations, which, while compelling, are preliminary in nature. The primary cellular targets of ALP—whether cardiomyocytes, macrophages, or fibroblasts—remain to be conclusively identified.

Therefore, future research will focus on several key directions: firstly, employing cell-type-specific knockout models to definitively establish the causal role of the TLR4/NF-κB pathway in different cardiac cell populations; and secondly, investigating other potential mechanisms, such as ALP’s reported vasorelaxant effects, to elucidate its impact on cardiac hemodynamics and overall functional benefit. These investigations will be crucial for fully validating ALP as a novel and targeted therapeutic candidate for ischemic heart disease.

## Figures and Tables

**Table 1:** Cardiac indices of rats in the three groups.

	Sham Group	AMI Group	AMI + ALP Group
Body weight (kg)	0.26 ± 0.02	0.27 ± 0.01	0.25 ± 0.01
Heart weight (g)	0.84 ± 0.08	0.94 ± 0.13	0.87 ± 0.05
Cardiac mass index (g/kg)	3.22 ± 0.14	3.56 ± 0.33	3.41 ± 0.20
LV weight (g)	0.53 ± 0.04	0.66 ± 0.06 ****	0.56 ± 0.02 ^##^
LV mass index (g/kg)	2.06 ± 0.04	2.42 ± 0.11 ****	2.21 ± 0.09 ^##^

LV, left ventricular; AMI, acute myocardial infarction; ALP, alpinetin (**** *p* < 0.0001 vs. Sham; ^##^ *p* < 0.01 vs. AMI). *n* = 6.

## Data Availability

The datasets generated during and/or analyzed during the current study are available from the corresponding author on reasonable request.
